# The demographic and morbidity characteristics of a population receiving food support in Israel

**DOI:** 10.1186/s13584-018-0238-8

**Published:** 2018-08-30

**Authors:** M. Endeweld, R. Goldsmith, R. Endevelt

**Affiliations:** 1 The Israel National Insurance Institute, Jerusalem, Israel; 2 The Hebrew University in Jerusalem, Jerusalem, Israel; 3 The Israel Ministry of Health, Nutrition Division, Jerusalem, Israel; 4 University of Haifa, School of Public Health, Haifa, Israel

**Keywords:** Food insecurity, Poverty, Food bank, Morbidity, Diabetes, Hypertension, Food support

## Abstract

**Background:**

Food supply to poor populations is a big challenge, particularly in periods of economic stress and in an era of chronic diseases epidemics. In Israel more than 4000 poor families are currently receiving food support. Many of the food support products given to the family have low nutrient values, therefore not appropriately ensuring food security of the population.

The aim of the current study was to examine for the first time the demographic, nutritional and chronic diseases profiles of food support beneficiaries, so as to aid in planning future food support components in Israel. In addition, the study examined associations between levels of food insecurity status and selected morbidities among food support recipients.

**Methods:**

In 2016, 3000 families (classified as very poor) in 24 municipalities received food support in Israel from the “National Food Security Project” (NFSP), under the guidance of the National Food Security Council. The 400 new families who joined the program in 2016 were requested to complete a questionnaire regarding the demographic and health characteristics of their families. Three hundred sixty-two of them completed the questionnaire for a response rate of 90%. The current study includes these families only.

**Results:**

The disposable income per capita of the surveyed families was very low – less than NIS 1100 a month ($280). About half the families were working families and 40% of them were in debt**.** Of the 362 responding families, about 82% of them were food insecure, with more than half severely food-insecure; this, despite receiving food support.

About one-third of the families had at least one member with anemia, and a quarter of the families had a member with hyperlipidemia. Hypertension is present in about 22% of the families, diabetes in 17%, and there is a 12% incidence of at least one family member with heart disease. These rates are markedly higher than those in the general population. Higher levels of food insecurity were associated with higher levels of hyperlipidemia, heart disease and hypertension.

**Discussion and conclusions:**

The nutrition and medical status of the population receiving food support is much worse than in the general population. There is a need to improve the nutritional value of food support; this could include greater emphasis on whole grains, fruits and vegetables. There is also a need for a nationwide education program to focus on healthy nutrition and to subsidize healthy foods. Many health and nutrition promotion models show that in order to effect changes in dietary habits and behaviors related to improving nutrition, there is a need for nutrition education (Kamp et al., J Nutr Educ Behav 42:72–82, 2010).

## Background

### Association between food insecurity and health

Food support plays a major role in the food aid sector by distributing donated and purchased groceries directly to food insecure families. The public health implications of food insecurity are significant, and food insecurity is more prevalent among certain population groups .Lack of appropriate nutrition education, coupled with food insecurity' may lead to poor food choices, which in turn, impacts upon health status [1, 2]. Hunger and poverty are linked to disease and malnutrition. Another hidden side of hunger and poverty is the higher prevalence nowadays of chronic diseases including those that are obesity related among the hungry and the poor [3].

Studies have shown the connections between food insecurity and morbidity.

Food insecurity was associated with higher HbA1c levels which did not improve over time [4, 5]. Depression, diabetes, distress and medication adherence are more connected to the food insecurity than to the level of poverty itself [6].

The link between economic and health status is well known and documented in many countries. More income and better education are linked to longer life expectancy and better health outcomes [7] (Cutler et al. 2011). Condiffe and Link (2008) [8] showed that there is more chronic disease among children in low income families. Food insecurity is positively correlated with the family’s economic status (NII, 2014).

In the USA, in the 2007–2008 National Health and Nutrition Examination Survey, food insecurity was measured using the United States Department of Agriculture (USDA) food security module. A sub sample included youth ages 4–17, living in families whose income is below 200% of the federal poverty line. It was found that (Body Mass Index) BMI z-scores indicating obesity were higher among participants in the food support programs than among the equivalent youth not receiving food support ( [9]). Similar results were seen in other countries (references). For example, a study done in 3 cities in Germany found that food support clients (*N* = 276, response rate of 21.5%) had similar socio-demographic characteristics (age, nationality, education, professional qualifications, household income) to those not receiving support with rates of smoking, having at least one chronic illness, estimating their own health status as moderate to poor and low consumption of fruits and vegetables similar in both groups In the low SES population the prevalence of diabetes was twice as high among food support clients and they also reported worse health status. Considerably lower fruit consumption and lower hypertension prevalence among females was noted, and lower overweight prevalence among male food bank clients were found [10].

Israel is similar to other countries in that those living in poverty and receiving food support over many years received food support which was intended to supplement caloric intake but not necessarily foods of high nutritional value. Partially as a result of these actions, the prevalence of chronic nutrition-related diseases in this population group has increased. The USA governmental programs described (SNAP, WIC) have recently changed the types of foods offered, so as to improve health outcomes.

### Food support programs

According to the National Households Survey carried out in 2012 by the National Insurance Institute in Israel (NII), 8.3% of households were classified as being moderately food insecure and 10.6% were classified as being severely food insecure [11]. In the absence of public policy interventions, the only immediate assistance available to households struggling to meet their food needs is charity, typically provided through food support. This is similar to Canada but different from the situation in many European countries and in the USA (United States of America) where mainly government programs take care of the needs of this population.

In the USA, three governmental nutrition assistance programs are in place, namely the Supplemental Nutrition Assistance Program (SNAP), the Special Nutrition Program for Women, Infants and Children (WIC), and the National School Lunch Program (NSLP) and these three programs serve there as the backbone of the nutrition safety net. These programs have been successful in achieving many of their initial goals of improving food purchases, food intake, and/or nutritional status of low-income, nutritionally vulnerable Americans. The emphasis in these programs has now broadened to focus also on obesity prevention with recent changes in program components reflecting these revised objectives. In a new study, Harnack et al. (2016) show that food benefit programs that pair incentives for purchasing more fruits and vegetables with restrictions on less nutritious foods, may reduce energy intake and improve the nutritional quality of the diet [12]. These changes were made in 2016, and as yet, an evaluation of the impact has not been carried out.

### In Israel

Several studies about food insecurity in Israel have been published, though none of them relate to the chronic disease situation of the food insecure population. One study (Phillip et al., 2016) has related to the technical aspects of food rescue in Israel [13]. Another study (Troen et al., 2006) compared nutrient intakes among food insecure Palestinian and Israeli families, with a focus on possible fortification, but did not relate to health status. Micronutrient intakes were unsatisfactory in both populations [14]. Two studies examined associations between psychiatric illness and food insecurity, and found high levels of food insecurity among the patients (2013, 2011) [15, 16]. A Food Security Survey carried out by the Brookdale Institute, in 2003 in conjunction with the Ministry of Health, found strong associations between levels of food insecurity and nutrients intake, both macro and micronutrients (https://brookdale.jdc.org.il/en/publication/food-security-israel-2003-implications-patterns-nutrition/).

In 2012 the Welfare Ministry launched the “National Food Security Project” (NFSP), under the guidance of the National Food Security Council. This project is operated by Eshel Jerusalem, which allocates monthly food baskets, valued at NIS (New Israeli Shekel) 300–600^1^ (depending on family size), to 3000 households in 24 municipalities. These municipalities were selected by the Ministry of Social Welfare from among municipalities with low SES levels. Within these municipalities, the program was offered to families with the lowest socio-economic status, and eligibility is means-tested. The program is able to fund a limited number of families, and due to changes in circumstances, residence etc., each year some families leave, and others join.

In Israel about 3000 families are supplied with dry foods once a month (see below).

Participating families are accompanied by the project coordinators and in some cities families are required to attend workshops and training groups on nutrition and home finances.

This project operates on the basis of the eligibility tests determined by Endeweld and Shmueli [17] (2013). These tests allow for granting of assistance to families whose income available for food, after deducting essential expenses on housing, electricity, water, gas, local property taxes, medications and some amount of debt, is less than the minimal amount needed for food, as defined by the National Insurance Institute, based on a study defining poverty based on expenditures. The project in its current form is focused primarily on families with children.

Due to the limited scope of the project at this stage, most of the participating families are amongst the poorest in Israel – that is, their net income per standard person falls by tens of percentage points below the official poverty line^2^.

In the framework of the research and assessment accompanying the project, participants were asked to fill out a questionnaire which examined the periodic changes in their food security level, their degree of satisfaction with the service provided, (both the food aspect and the workshops in which some of them participate), recommended improvements, etc.

The aim of the current study was to examine for the first time the demographic, nutritional and chronic diseases profiles of food support beneficiaries so as to aid in planning future food support components in Israel, so as to improve chronic diseases profiles as needed. In addition, the research aimed to examine whether the level of food insecurity of families living in severe poverty enrolled in the food support program are associated with differences in selected morbidities.

## Methods

In 2016 all of the 400 new families joining the National Food Security Program (NFSP) were requested to complete the questionnaire which included a health component- their completion of the questionnaire being a prerequisite for entry into the program. three hundred sixty-two agreed, for a response rate of 90%. The socio-demographic profiles of these “new” families is not different from those previously enrolled, thus validating the representativeness of this study. This is based on a comparison, done to ensure homogeneity, of the socio-demographic profiles of the newly-enrolled participants (as determined from eligibility tests as part of enrolment) with the socio-demographic profiles of existing participants - done to ensure homogeneity.

Beginning in 2016 a new questionnaire was distributed to participants. Included in this questionnaire, besides socio-demographic questions, questions related to health and questions related to the level of satisfaction with the services provided to them by the NFSP, was a new section - the Six-Item Short Form of the 18-item U.S. Household Food Security Survey Module [18]. This six-item module, found to be an acceptable substitute for the 18-item module, can identify food-insecure households and households with very low food security with reasonably high specificity and sensitivity and minimal bias compared with the 18-item measure. It does not, however, directly ask about children’s food security, nor does it measure the most severe range of adult food insecurity, in which children’s food intake is likely to be reduced. A full description of the Food Security Module is included in Appendices 1 and 2.

The Six-Item Short Form questionnaire, to which 362 families responded (the “respondent” being one of the adults in the family, usually the mother) is the data source of the current research.

In addition, the new questionnaire included five questions on family morbidity, developed by researchers at the National Center for Health Statistics in collaboration with Abt Associates Inc [19, 20]. The aim was to ascertain whether someone in the family (regardless whether an adult or child) had one of the following conditions: anemia, hyperlipidemia, diabetes, heart disease or hypertension. This was based on self- report. Though data on obesity was not collected, the causal association between diabetes, hypertension, heart disease and obesity has been well established.

It should be noted that besides the limitation that these questions did not differentiate between adults or children, in addition they provide no information on the prevalence of the condition/s within the family (see also “Limitations” below).

In order to examine the association between food security and morbidity, logistic equations were estimated, where the dependent variable was the illness/condition, and the dependent variable relating to food security was estimated in two ways: (1) existence of food insecurity; (2) scores for the family (as described above).

Additionally included in the vector were the independent variables –demographic characteristics (nationality, age, religiosity, etc.) and economic variables (income per capita, receiving disability pension or income support, etc.), gathered both from the questionnaire above and other information received from families participating in the project and other tools. Thus, for example, we used financial data from the eligibility test, which includes information about income, expenses and family debt.

The analyses of associations between food security and chronic disease were not carried out to test whether food security is a cause of chronic illness; this would not have been possible given the cross-sectional, single point in time, nature of the study. Rather, these associations were analyzed undertaken primarily to assess the extent of significant health challenges among persons experiencing food insecurity, so that these challenges can be taken into account in the design of food assistance programs.

## Results

### Characteristics of respondents

Respondents’ population segmentation by demographic, socio-economic, morbidity and food security variables are shown in Table 1 below. The data show the characteristics of the 362 families who responded to the repeat questionnaire. They are a representative cross-section of the families participating in the food security project, as described in the summary report of the National Food Security Survey (Endeweld, M et al.)^3^. Data from this project as well as data from the recent National Food Security Survey of the National Insurance Institute is currently in preparation towards publication.

It can be seen that only 12% of the respondents are Arab families, a slightly lower rate than in the general population (14.6% [21]). Among Jewish families, one quarter is ultra-Orthodox families (a rate higher than in the general population (6.4%), but reflecting the high representation of this population group among the poor in Israel) and about one quarter is secular families (a lower percentage than in the general population). The remainder, about half of the Jewish families, are religious or traditional, thus there is a greater concentration of religious families in the sample than in the general population, where the rate is 14%. This explains how the average number of persons per family reaches the high level of 5.25 (compared to 3.3 in the general population according to Households Surveys of the Central Bureau of Statistics in the recent years).

The disposable income per capita is very low – less than NIS 1100 a month. About half the families are working families and 40% of them are in debt to various entities, such as banks, municipalities (city taxes), charity organizations, etc. About two-thirds of respondents are female and in the study sample there is a greater concentration of single-parent families (only half of the respondents are married) than in the general population (5.7%).

Of the 362 families, about half received some^4^ food support before joining the project, but a very small proportion – only 7% – regularly received this assistance, namely at least once a month (Table 2).

The food security situation of the respondent families is very difficult, as to be expected: about 82% of them are food insecure, with more than half severely food-insecure [as defined by the score on the HFSS 6 item questionnaire. It should be noted that, according to one interpretation, even a score of 1 (out of 6) indicates moderate food insecurity. Taking into account the families who received this score (see the table of scale score below), it turns out that all had some level of some food insecurity (none received a score of zero). The average score of the Food Insecurity Rasch Scale is 6.4 (out of a total of 8.5 points) among respondents’ families.

The following table shows the scores distribution.

**Table:** 

Num. of positive answers	grade	Freq.	Percent
1	2.86	65	17.96
2	4.19	37	10.22
3	5.27	27	7.46
4	6.3	36	9.94
5	7.54	54	14.92
6	8.48	143	39.5
Total		362	100

It should be noted that these levels of food insecurity existed while these families were participating in the NFSP. According to all indices developed during the research, the project had a significant positive impact on the food security status of the participating families. That is, the situation would have been much worse if these families had not participated in the project.

Examination of morbidity data shows a relatively high incidence of illness/conditions in the families. About one-third of the families have at least one member with anemia, and a quarter of the families have a member with hyperlipidemia. Hypertension is present in about 22% of the families, diabetes in 17%, and there is a 12% incidence of at least one family member with heart disease. About 55% of the families have at least one of the five diseases/conditions indicated in the questionnaire. These are well above the rates in the general population, in particular for heart disease, at 4.6% and diabetes., at 7.7% [22].

Table 3 below indicates the scores and levels of food insecurity by population groups. The results show no difference in the severity of food insecurity between Arabs and Jews participating in the project. Characteristics of the families of both groups were similar, though Arab families were relatively younger: the average age of Arab respondents was 39.6 compared to 42.9 of Jewish respondents. Unmarried families (single parent) receiving income support have more severe food insecurity than married families, and among the Ultra-orthodox families food insecurity was more moderate.

**Table 1:** Socio-demographic characteristics (*N* = 362)

		Percent	Mean (sd)
Age of responder (years)			42.5 (10.4) (Range-21-77, 85% in range 25–55)
Average age (of respondents)	Jewish		42.9 (9.5)
	Arab		39.6 (10.5)
Household density (no. of persons)			5.25 (2.4)
Average no. of children			3.1
Ethnicity	Jewish	88	
	Arab	12	
Religious level (Jews only)	Ultra Orthodox	22	
	Orthodox (religious)	12	
	Traditional	34	
	Secular	21	
Marital status	Married	50	
	Single (including divorced, widowed, and single parents- all with children	50	
Receiving food support	Yes, before joining NFSP	50.6	
	Yes, regularly	7.7	
Food security status Of which	Food insecure	82.0	
	Severely food insecure	54.4	
Food insecurity score			6.44 (2.2)
Net per capita monthly income	In ILS		1086 (568.5)
Own a home			50%
Working households		48	
Weekly hours of work			38.1) 26.6)
Receive benefits	Disability	34	
	Income support	37	
Debts	Household with debts	40	
	Average debt (NIS)		32,330 (104,093)

**Table 2:** Morbidity characteristics (*N* = 362)- of at least one household member

	% in household
Anemia	34.8
Hyperlipidemia	25.7
Diabetes mellitus	17.4
Heart Disease	11.9
Hypertension	21.8

**Table 3:** Food Insecurity Scale Score of population groups

		Average score	Distance from average for the full sample (%)
Total population		6.4	
Household density (no. of persons)	Up to 4 people	6.6	3
	5 or more	6.3	-2
Marital status	Married	5.9	−8
	Single	6.9	8
Ethnicity	Jewish	6.4	0
	Arab	6.4	0
Religious level (Jews only)	Ultra-Orthodox	5.6	−13
	Other	6.7	5
Benefits	Disability	6.4	0
	Income support	6.9	8
Debt	Household has debt	6.3	−2

### Chances of morbidity by food security state

In order to examine the factors associated with the likelihood of morbidity from the five diseases/conditions investigated, we estimated the logistic regression model in two forms, with the dependent variable in both forms being the presence or absence of selected diseases/conditions in the family. As noted, food security variables were of two types (1) the existence of food insecurity (2) scores for the family. Independent variables include demographic and socio-economic variables as seen in Tables 4 and 5 (in Appendices 3 and 4), showing the ratios of morbidity for each of the diseases.

The results show that in the two models estimated (see details in Appendices 3 and 4), when dealing with a very low socio-economic cross-section of the population enrolled in a food support program, the level of food insecurity is associated with levels of hyperlipidemia, heart disease and hypertension. In the second model, when the independent variable is not categorical but allows for intervals between families, the results are of higher statistical significance and the impact of the food insecurity level among very low income families is significant also for diabetes (despite the relatively low significance level: only 0.1). Results show that there is a direct correlation between an increase in the level of food insecurity, as assessed by the score on the Food Insecurity Scale, and increased risks of presence of four of the five diseases/conditions reported. For every one point increase in the scale the risk of hyperlipidemia, diabetes, heart disease and hypertension increase by 20, 16, 31 and 21%, respectively. The first model, with the categorical variable of food insecurity, shows that the state of food insecurity is associated with a high risk for morbidity in the family. When comparing the prevalence of chronic diseases as shown above with prevalence reported for the general population in Israel (The State of Health in Israel) it can be seen that the prevalence (among the food insecure) are more than 2.5 times higher for diabetes, at 17% as compared to 7.7% in the general population and more than 4 times for anemia, at 30% compared to 6–7% in the general population [22].

It was also found that in very low-income families who enrolled in a food support program, the income per capita and existence of debt do not affect morbidity. There is a strong (as expected) relationship between morbidity and receipt of disability benefits from the National Insurance Institute (but no significant correlation with income support). Among Jews in general there are greater chances of higher morbidity than among Arabs and there is lower morbidity among ultra-Orthodox than among other Jewish population groups. Each additional year in the age of respondents increases the chances of morbidity from 5 to 9%, and the marital status “married” significantly increases the chances of diabetes in the family.

**Table 4:** Logistic regression results – Odds ratios- for morbidity from five diseases*; as the food security independent variable 1- is family living with food insecurity

	Anemia	Hyperlipidemia	Diabetes mellitus	Heart disease	Hyper-tension
Household with food insecurity	0.97	2.43^**^	1.82	4.65^**^	3.06^**^
Age	0.99	1.06^***^	1.09^***^	1.05^***^	1.09^***^
Married	0.74	1.42	3.31^***^	1.43	1.31
Jew	3.60^***^	1.68	2.31	(omitted)	1.2
Ultra-Orthodox	0.39^***^	0.49	0.57	0.41	0.61
Num. of persons in household	1.02	1	1	1	1.04
Receive disability benefits	1.89^**^	4.16^***^	4.09^***^	1.25	2.46^***^
Receive income support	1.03	0.62	0.74	1.14	1.11
In debt	1.21	1.08	0.87	0.95	0.67
Income per capita	1.00	1.00	1.00	1.00	1.00
Statistics					
N	362	362	362	319	362
chi^2^	24.74	76.72	76.08	23.66	68.91

Anemia, Hyperlipidemia, Diabetes, Heart disease, Hypertension

legend: ^*^ *p* < .1; ^**^ *p* < .05; ^***^ *p* < .01

**Table 5:** Logistic regression results-Odds Ratios- for morbidity from five diseases/conditions(listed below) as the independent variable 2- Food Insecurity Scale Score

	Anemia	Hyper-lipidemia	Diabetes mellitus	Heart disease	Hyper-tension
Food insecurity scale score	1.05	1.20^***^	1.16^*^	1.31^***^	1.21^**^
Age	0.99	1.06^***^	1.08^***^	1.05^***^	1.09^***^
Married	0.78	1.55	3.70^***^	1.65	1.42
Jew	3.60^***^	1.54	2.17	(omitted)	1.09
Ultra-Orthodox	0.41^**^	0.46^*^	0.54	0.36^*^	0.56
Num. of persons in household	1.02	1.01	1	1.02	1.06
Receive disability benefits	1.90^**^	3.96^***^	3.95^***^	1.15	2.32^***^
Receive income support	0.99	0.60^*^	0.73	1.12	1.1
In debt	1.2	1.04	0.86	0.91	0.65
Income per capita	1.00	1.00	1.00	1.00	1.00
Statistics					
N	362	362	362	319	362
chi^2^	25.57	79	77.84	25.72	69.7

Anemia, Hyperlipidemia, Diabetes, Heart disease, Hypertension

legend: ^*^p < .1; ^**^ *p* < .05; ^***^ *p* < .01

## Discussion

The aim of this study was to examine for the first time the connection between the levels of food insecurity and selected morbidities by describing the profiles of 362 households of the 4000 participating in the “National Food Security Project” (NFSP). These 362 families responded to a questionnaire specifically designed to determine the level of satisfaction of the households, the level of food insecurity (according to the six-item module of the Household Food Security Survey) and information about selected morbidities in these households. In particular, the respondents (usually one of the adults) answered whether someone in the family (regardless of whether adult or child) had one or more of the following conditions: anemia, hyperlipidemia, diabetes, heart disease or hypertension.

The results show that for every additional point in the food insecurity scale score the risk of hyperlipidemia, diabetes, heart disease and hypertension increases by 20, 16, 31 and 21%, respectively within the families who receive food support. These compare to much lower prevalence of these diseases/conditions in the general population in Israel. Among Jews in the study there is greater prevalence of morbidity than among Arabs. The biggest difference between Jews and Arabs was found in the probability of anemia, which is 3.6 times higher among Jews as compared with Arabs. This may be attributed to meat consumption habits of Arab families. There is also lower morbidity among the ultra-Orthodox as compared to other Jewish population groups. This may be explained by different lifestyle habits not related to nutrition habits, such as lower levels of stress and anxiety, and a more supportive community. It is also possible that the range of ages is lower in Ultra Orthodox households, and the respondent’s age component (which is part of the vector of explanatory variables in the regression) did not completely control for the full effect of age.

An additional significant finding is the difference in prevalence of diabetes which is much higher in families where heads of households were married as compared to single head of households. The finding may be partly explained by the nature of food purchasing for larger families, in that the necessity to purchase large food quantities on a limited budget often results in selection of cheaper foods of lower nutritional value, thus resulting in increased prevalence of diseases/conditions such as diabetes and hyperlipidemia, which are partly attributable to poor dietary habits. In addition, purchasing in bulk may reduce the degree to which the quality of the food.is checked.

These results can be seen, with the limitations of the comparisons, in other countries such as the USA.

There is no doubt regarding the importance of the programs but could they be improved, in order to give that population the right food with the right educational programs?

In the USA, programs for nutritional assistance -Supplemental Nutrition Assistance Program (SNAP), Special Nutrition Program for Women, Infants and Children (WIC), and National School Lunch Program (NSLP)-serve as the backbone of the nutrition safety net in the USA.

These programs have been successful in achieving many of their initial goals of improving food purchases, food intake, and/or nutritional status of low-income, vulnerable Americans. The only goal that was not achieved yet was the health benefit and obesity prevention, so as to prevent the resultant increased risk of chronic disease development [23].

A few studies done on the SNAP program found that the program did not address the problem of obesity and may even contribute to the problem [24].This may be as a result of the lack of regulation, until recently, regarding the type of food that could be purchased. As a result, often nutritionally poor foods were purchased, which in turn may have contributed to higher prevalence of diseases and conditions known to be related to poor dietary habits. In the last year, regulations have been introduced to define which foods may be purchased, with the emphasis on healthier nutritionally sound foods. Another problem that was found is the very low literacy level of the population receiving the program wherein low literacy is associated with poverty and economic disparities [25].

There have been many papers published investigating the correlation between food assistance programs and obesity and chronic disease. It appears that the correlation depends on age, gender and the specific food assistance program. For example, it was found that participants in the Supplemental Nutrition Assistance Program (SNAP) were more likely than nonparticipants (of equal or higher income) to be obese [26]. However, this correlation appears to be gender-specific. Current and long-term participation in the food stamp program was significantly correlated with obesity in low-income women, but the association did not exist among men [27]. Also the specific assistance program impacted on the correlation. In preschool children, research showed that children from lower-income families had higher incidences of obesity, hypertension, and dyslipidemias [28]. And while participation in SNAP and the national school lunch program (NSLP) appeared to be correlated with certain cardiovascular risk factors, participation in the Special Nutrition Program for Women, Infants, and Children (WIC) may actually be beneficial in combating these cardiovascular risk factors [12]. In research conducted in Peru, it was shown that children participating in the Glass of Milk program had a 65% lower risk of obesity than those not participating. For mothers, however, participants in the Community Kitchens program had twice the risk of obesity when compared to nonparticipants [29].

A current study published by a Portuguese group show that when examined the impact of food insecurity on health and health-related issues they found that subjects with food insecurity reported worse quality of life score HRQoL and more physical disability when compared to subjects without food insecurity [30]. A higher proportion of subjects with food insecurity were found to have diabetes and rheumatic diseases than those with food security. In fact, their results agree with those from other countries that found strong evidence that vulnerable people, who commonly live in food insecure conditions, have a higher risk of poor health [31]. Studies have found that socioeconomically vulnerable groups experience higher mortality and morbidity rates for coronary heart disease [32], atherosclerosis, type 2 diabetes mellitus (34), and some cancers [33]. The Portuguese study also revealed that a high proportion of subjects with food insecurity reported mental illness in the form of self-reported depression symptoms. The consequences of food insecurity in mental health, namely anxiety and depression symptoms, are well documented in the literature [34].

The possible correlation between food assistance programs and obesity and chronic diseases may have to do with the diet quality of the participants.

Among adults with obesity and diabetes, food insecurity is associated with lower overall dietary quality, increased consumption of unhealthy foods and lower consumption of healthy foods. These individuals are limited to cheaper, more calorie-dense and high-sugar foods that promote high blood sugar [35].

Data show that SNAP participants received a larger percentage of their calories from empty calories (solid fats, added sugars) when compared to nonparticipants [10]. Overall, Healthy Eating Index− 2005 scores for SNAP participants were lower than those of nonparticipants [10]. Participation in WIC and the NSLP, however, was correlated with increased nutrient quality without increased caloric intake [36]. Policy revisions for all the food assistance programs now aim to improve diet quality. For SNAP, incentivizing the purchase of nutrient-dense items appears to be beneficial (https://brookdale.jdc.org.il/en/publication/food-security-israel-2003-implications-patterns-nutrition/). It has been suggested that limiting the choices of foods that SNAP participants receive would be beneficial, but the implementation is complicated (https://brookdale.jdc.org.il/en/publication/food-security-israel-2003-implications-patterns-nutrition/). Overall, it is understood that nutrition education must be implemented to improve diet quality of SNAP participants as is done in the Israeli programs.

In a study that was conducted among 279 lower income participants not enrolled in SNAP program, Harnack et al. (2016) wanted to evaluate whether prohibiting the purchase of less nutritious foods with SNAP benefits improves the nutritional quality of foods purchased and consumed. It was found that a food benefit program that pairs incentives for purchasing more fruits and vegetables with restrictions on the purchase of less nutritious foods may reduce energy intake and improve the nutritional quality of the diet of participants compared with a program that does not include incentives or restrictions.

The WIC program, with estimated expenditures of $6.8 billion annually and its authorized vendors, serves approximately 8.9 million infants, children, and pregnant/postpartum women each month. This population also faces the problem of an increased prevalence of obesity. In 2009 fruits and vegetables were added to the program to try to contain the obesity epidemic which is common among lower socioeconomic status women in the United States. This action resulted in lower prices of fruit and vegetables in small stores where those women tend to buy their food products [37]. Improving the quality of foods in the food stores for the WIC program changed the purchase patterns to healthier ones. The food guidelines included more fruit and vegetables and whole grains [38]. Changes to the food packages offered by WIC (such as fruit and vegetable vouchers) aim to improve diet quality [10]. These policy changes may also affect vendor prices for fruits and vegetables, which further encourages their consumption [19]. In Israel this was not part of the program.

Finally, new regulations for the NSLP aim to improve nutrient intake [13]. Though a correlation between food assistance programs, such as SNAP and NSLP, and obesity appears to exist, evidence is not sufficient for such a correlation to be proven (https://brookdale.jdc.org.il/en/publication/food-security-israel-2003-implications-patterns-nutrition/).

Because of the results of these previous studies, this year these programs are aiming to address the obesity and resulting chronic disease problem and to change the quality of the food products and the environment and education to allow for better nutrition habits and status.

### Limitations

The methodology and the questionnaire did not provide morbidity data on each household member, and it is possible that less morbidity was correlated to a younger household composition (family with many young children) rather than other demographic characteristics.

The obesity information is lacking because the questionnaire did not directly examine this. Unlike questions on the other disease states which did not specifically relate to the respondent but rather to the incidence within the family, questions on weight and height are of necessity person-specific and may have met with an antagonistic response, because of perceived stigmatization. Therefore, questions on weight/height and obesity were not included. It is recognized that this issue is very important and future research of this nature will attempt to explore obesity prevalence in a non-intrusive manner.

There is adequate representation of Arab families in the sample (12%) as compared to 13% in the general population. However, about 30% of Arab families suffer from food insecurity, thus were in fact underrepresented in the sample. As the initial stages of the proposed new initiative were in the central areas of the country, and many Arab villages and towns are in the periphery, they were underrepresented. However, the number of Arab families included is large enough to allow for statistical comparisons.

In addition, there may be municipalities which, for various reasons, do not participate in the National Food Security Project and their health and socio-demographic characteristics may be different.

## Conclusions

The low socioeconomic population in Israel suffers more from obesity and diabetes and heart diseases: the incidence of diabetes in the general population is 9.7% but in the low socioeconomic population it has reached 25% [39] (like in countries such as the USA and others, where nutrition support program for mother and child also exist and there it can be also seen that the mothers suffer much more from obesity than the general population in the country [40, 41]). Since the nutrition and medical status of the population receiving food support is worse than that of the general population, there is a need not only to expand the food support to many more families, but also to change the food support to a healthier one. The composition of the food support should be under the supervision of the Nutrition Division of the Ministry of Health, and the Division should be involved in the monitoring alongside that of the Welfare Ministry. Prior approval of food support contents, with the aim of maximizing nutrient contents and benefits, should be required (preferably mandated) to be given by the Nutrition Division. In addition, a nationwide education program on healthy nutrition and subsidizing healthy foods would be effective policy steps, not only for the poor and those in need of food support services, but also for the entire population.

## Appendix 1

### Six-Item questionnaire distributed to the NFSP participants*

1. I’m going to read you several statements that people have made about their food situation. For these statements, please tell me whether the statement was often true, sometimes true, or never true for (you/your household) in the last 12 months—that is, since last (name of current month).

The first statement is, “The food that (I/we) bought just didn’t last, and (I/we) didn’t have money to get more.” Was that often, sometimes, or never true for (you/your household) in the last 12 months?

[] Often true

[] Sometimes true

[] Never true

2. “(I/we) couldn’t afford to eat balanced meals.” Was that often, sometimes, or never true for (you/your household) in the last 12 months?

[] Often true

[] Sometimes true

[] Never true

3. In the last 12 months, since last (name of current month), did (you/you or other adults in your household) ever cut the size of your meals or skip meals because there wasn’t enough money for food?

[] Often true

[] Sometimes true

[] Never true

4. How often did this happen—almost every month, some months but not every month, or in only 1 or 2 months?

[] Almost every month

[] Some months but not every month

[] Only 1 or 2 months

5. In the last 12 months, did you ever eat less than you felt you should because there wasn’t enough money for food?

[] Often true

[] Sometimes true

[] Never true

6. In the last 12 months, were you every hungry but didn’t eat because there wasn’t enough money for food?

[] Often true

[] Sometimes true

[] Never true

* Minor changes were introduced compared to the USA six item module: instead of answers “yes”/“No” in questions 3,6, and 6 we continued the form of “often true/sometimes true/never true” (the first two considered as “positive”). In addition, we did not allow for “DK” (don’t know) answers.

## Appendix 2

### The six-question food security module

In the 6 item version of the survey, one point is given for every affirmative/positive answer. (Responses of “often” or “sometimes” on questions 1,2,3,5,6, and responses of “almost every month” and “some months but not every month” on question 4 - see Appendix 1), so that the maximum number of points is 6.

Food security status is assigned as follows:

- Raw score 0–1 — High or marginal food security (raw score 1 may be considered marginal food security, but a large proportion of households that would be classified as having marginal food security using the 18 item household or adult scale will have a raw score of zero on the six-item scale).
- Raw score 2–4 — Low food security;
- Raw score 5–6 — Very low food security.

For statistical procedures that require an interval-level measure, the following scale scores, based on the Rasch measurement model, may be used, allowing one to examine quantitative differences among the various food security levels in addition to the categorical form described below.

The Rasch model provides a framework in which to simultaneously estimate the individual abilities and the item difficulty parameters, based on a set of questions administered to a group of individuals (see Baker, 1992) [42].

**Table:**

Scale score	Number of affirmatives
NA	0
2.86	1
4.19	2
5.27	3
6.30	4
7.54	5
8.48	6 (evaluated at 5.5)

## Abbreviations

BMI: Body Mass Index
NFSP: National Food Security Project
NII: National Insurance Institute in Israel
NIS: New Israeli Shekel
NSLP: National School Lunch Program
SNAP: Supplemental Nutrition Assistance Program
USA: United States of America
USDA: United States Department of Agriculture
WIC: Special Nutrition Program for Women, Infants and Children

## Footnotes

1 To get an idea, the average expenditure on food including fruit and vegetables for household in 2014 was about 2400 NIS.

2 The official poverty line is determined in Israel, like in most Western countries and in the OECD, according to a relative method: as half the net income per standard person.

3 Endeweld, M., (2012–2015) Summarizing reports of National Food Security Project (in Hebrew), mimeo.

4 We do not have information as to whether those accepted received food support in the past. One can assume that if so, they are only a small number, and it is safe to assume that this project is their main form of food support.
